# sFlt-1 Is an Independent Predictor of Adverse Maternal Outcomes in Women With SARS-CoV-2 Infection and Hypertensive Disorders of Pregnancy

**DOI:** 10.3389/fmed.2022.894633

**Published:** 2022-05-09

**Authors:** Jose Antonio Hernandez-Pacheco, Johnatan Torres-Torres, Raigam Jafet Martinez-Portilla, Juan Mario Solis-Paredes, Guadalupe Estrada-Gutierrez, Paloma Mateu-Rogell, Miguel Angel Nares-Torices, Mario Enmanuel Lopez-Marenco, Keren Rachel Escobedo-Segura, Alejandro Posadas-Nava, Jose Rafael Villafan-Bernal, Lourdes Rojas-Zepeda, Norma Patricia Becerra-Navarro, Manuel Casillas-Barrera, Mauricio Pichardo-Cuevas, Cinthya Muñoz-Manrique, Ivan Alonso Cortes-Ramirez, Salvador Espino-y-Sosa

**Affiliations:** ^1^Clinical Research Branch, Instituto Nacional de Perinatología Isidro Espinosa de los Reyes, Mexico City, Mexico; ^2^Hospital de la Mujer, Mexico City, Mexico; ^3^Hospital General de Mexico Dr. Eduardo Liceaga, Mexico City, Mexico; ^4^Iberoamerican Research Network in Obstetrics, Gynecology and Translational Medicine, Mexico City, Mexico; ^5^Centro de Investigación en Ciencias de la Salud, FCS, Universidad Anahuac México Campus Norte, Huixquilucan, Mexico; ^6^Laboratory of Immunogenomics and Metabolic Diseases, Instituto Nacional de Medicina Genomica, Mexico City, Mexico; ^7^Maternal Fetal Medicine Department, Instituto Materno Infantil del Estado de Mexico, Toluca, Mexico

**Keywords:** endothelial dysfunction, preeclampsia, placental growth factor, soluble fms-like tyrosine kinase-1, SARS-CoV-2, COVID-19

## Abstract

**Background:**

Preeclampsia (PE) and COVID-19 share a common vascular–endothelial physiopathological pathway that may aggravate or worsen women's outcomes when both coexist. This study aims to evaluate the association of sFlt-1 levels and adverse maternal outcomes among positive SARS-CoV-2 pregnant women with and without hypertensive disorders of pregnancy (HDP).

**Methods:**

We performed a multicenter retrospective cohort study of pregnant women with confirmed SARS-CoV-2 infection that required hospital admission. The exposed cohort comprised women with a diagnosis of an HDP. The primary outcome was a composite definition of adverse maternal outcome. The association between predictors and the main and secondary outcomes was assessed using an elastic-net regression which comprised a Lasso and Ridge regression method for automatic variable selection and penalization of non-statistically significant coefficients using a 10-fold cross-validation where the best model if automatically chosen by the lowest Akaike information criterion (AIC) and Bayesian information criteria (BIC).

**Results:**

Among 148 pregnant women with COVID-19, the best predictive model comprised sFlt-1 MoMs [odds ratio (OR): 5.13; 95% CI: 2.19–12.05], and HDP (OR: 32.76; 95% CI: 5.24–205). sFlt-1 MoMs were independently associated with an increased probability of an adverse maternal outcome despite adjusting for HDP.

**Conclusions:**

Our study shows that sFlt-1 is an independent predictor of adverse outcomes in women with SARS-CoV-2 despite hypertension status.

## Introduction

Coronavirus disease 2019 (COVID-19) is caused by severe acute respiratory syndrome coronavirus 2 (SARS-CoV-2) (1). Pregnant women have a higher risk of severe COVID-19 (2, 3) caused by higher levels of inflammatory markers (IL-6, TNF-alpha, IFN-gamma, etc.), hypertension, oxidative stress, and thrombotic events causing multiorgan and placental damage with adverse outcomes for the mother and the developing fetus (4–7). Preeclampsia (PE) is defined as hypertension and proteinuria that debuts after 20 weeks gestation (8); its physiopathological pathway is similar to that of COVID-19, with an exaggerated inflammatory response that leads to endothelial damage and cytokine release from mesenchymal stromal cells of preeclamptic placentas (9, 10). SARS-CoV-2 infection is a significant risk factor for preeclampsia in pregnancy. In the participating hospitals, a substantial increase in preeclampsia is observed among women with pregnancy complicated by SARS-CoV-2 infection compared to women with a negative test (National Institute of Perinatology: 18 vs. 9%, General Hospital of Mexico: 12 vs. 5%). At a molecular level, there is an interaction between COVID-19 and PE, characterized by altered levels of angiogenic markers soluble fms-like tyrosine kinase (sFlt-1) and placental growth factor (PlGF) and higher sFlt-1/PlGF ratio (6, 11–13). Serum sFlt-1 is an antiangiogenic protein released from many tissues that antagonizes serum PlGF and vascular endothelial growth factor [VEGF, promoting endothelial dysfunction (14)]. Thus, PE and COVID-19 share a vascular–endothelial physiopathological pathway that may aggravate or worsen women's outcomes when both coexist. We hypothesize that this synergic effect is exemplified in sFlt-1 levels among PE and COVID-19 and that there may be an interaction between hypertensive disorders of pregnancy (HDP), COVID-19, and sFlt-1 levels that is reflected in the incidence of adverse maternal outcomes (15). Therefore, this study aims to evaluate the association between sFlt-1 levels and adverse maternal outcomes among positive SARS-CoV-2 pregnant women controlled by HDP.

## Methods

### Study Design and Participants

This is a multicenter retrospective cohort study of pregnant women with confirmed SARS-CoV-2 infection that required hospital admission at the Instituto Nacional de Perinatologia Isidro Espinosa de los Reyes, the Hospital de la Mujer, and the Hospital General de Mexico Dr. Eduardo Liceaga. All women with a confirmed SARS-CoV-2 infection by RT-qPCR test that required hospital admission were included. The exposed cohort comprised women with a diagnosis of an HDP that included preeclampsia, eclampsia, and HELLP syndrome according to ACOG criteria (16), between October 2020 and December 2021. Controls were those hospitalized SARS-CoV-2 pregnant women without hypertension in pregnant. The protocol was approved by the Ethics and Research Internal Review Board of the National Institute of Perinatology (2020-1-32) and was conducted ethically under the World Medical Association Declaration of Helsinki. All enrolled women authorized and provided signed informed consent.

### Data Collection

The following data were collected from the medical records: age, gestational age at admission, pregestational body mass index (pBMI) (kg/m^2^), mean arterial pressure (MAP) at admission, and history of previous diseases such as chronic hypertension, pre-gestational diabetes, preeclampsia, and pneumonia. Participants' blood samples were obtained at hospital admission as routine testing, and laboratory results were obtained: platelets, aspartate aminotransferase (AST), alanine aminotransferase (ALT). The blood sample for sFlt-1 and PlGF measurements was also drawn at admission. The time between SARS-CoV-2 diagnosis and hospital discharge.

### Measurement of Angiogenic Markers sFlt-1 and PlGF

A blood sample was drawn and immediately centrifuged (1,000 × g/10 min), plasma was aliquoted and stored at −70°C until analysis. Then, the plasma concentration of angiogenic markers PlGF (Elecsys PlGF, Roche^®^) and sFlt-1 (Elecsys sFlt-1, Roche^®^) were quantified by electrochemiluminescence using an automated analyzer (Cobas-e411, Roche^®^) according to the manufacturer's instructions. Biomarkers were log-transformed and converted to their multiples of the median (MoM) (17, 18) according to the Fetal Medicine Foundation algorithms (19).

### Outcomes

We constructed a composite endpoint of adverse maternal outcomes that comprised any of the following: (1) Pneumonia (according to the American Thoracic Society criteria). (2) Acute kidney disease is defined as a 50% increase in serum creatinine (sCr) concentration over baseline or a 0.3 mg/dL increase from the lowest value within 48 h or oliguria, which corresponds to KDIGO (Kidney Disease: Improving Global Outcomes) stage 1 AKI or higher (20). The baseline sCr level was defined as the lowest sCr measurement within the previous 7 days or the median of all outpatient sCr values obtained within 7–365 days before hospitalization (21, 22). (3) Multiple organ failure (defined as the alteration of two or more organs with a score of ≥3 according to Sequential Organ Failure Assessment (SOFA) Score) (23). (4) Maternal sepsis (defined as the presence of suspected or confirmed infection plus signs of mild to moderate organ dysfunction (e.g., tachycardia, low blood pressure, tachypnoea, altered mental status, reduced urinary output) (24). (5) Maternal death, (6) preterm rupture of membranes, or (7) threatened preterm labor. The secondary outcome was the composite of adverse fetal and neonatal outcomes that comprised the following: prematurity, small-for-gestational-age neonate, fetal growth restriction, cesarean section due to fetal distress, low Apgar score, Grade IV intraventricular hemorrhage, respiratory distress syndrome, neonatal sepsis, neonatal death, admission to the neonatal intensive care unit.

### Statistical Analysis

Groups were divided into an exposed and non-exposed cohort. Continuous variables were analyzed using the U-Mann Whitney test and expressed as the median and interquartile range (IQR); categorical data were analyzed using Chi-square or Fisher's exact test and expressed as number and percentage. The association between predictors and the primary and secondary outcomes was assessed using an elastic-net regression comprised of Lasso and Ridge regression for automatic variable selection and penalization of non-statistically significant coefficients using 10-fold cross-validation. The best model is automatically chosen by the lowest Akaike information criterion (AIC) and Bayesian information criteria (BIC). Predictive probabilities of an adverse outcome were calculated for the multivariate model adjusted by possible confounders selected by the elastic-net regression using the interaction between sFlt-1 concentration and hypertensive disorders of pregnancy (HDP) in a full factorial model for graph purposes. *P*-values <0.05 were considered statistically significant. (StataCorp. 2020. Stata Statistical Software: Release 17. College Station, TX: StataCorp LLC.).

## Results

### Description of the Cohort and Characteristics of the Study Population

One hundred forty-eight women with a positive RT-qPCR test for SARS-CoV-2 infection admitted to the hospital were included, of which 56 (37.8%) had an HDP. The mean maternal age was 30 (range: 15–45) years. When we compared by high blood pressure group in pregnancy, there were significant differences in mean arterial pressure (MAP), platelet count, ALT, AST, troponin, myoglobin, sFlt-1 MoMs, and sFlt/PlGF ratio (Table 1). Women with HDP had a higher incidence of pneumonia, acute kidney injury, lower birth weight, and a composite of adverse maternal outcomes (Table 2).

**Table 1:** Characteristics of the included population.

	**SARS-CoV-2 infection**		
**Characteristics**	**Controls**	**HDP**	***p*-value**
	**(*n* = 92)**	**(*n* = 56)**	
	**Median (IQR)**	**Median (IQR)**	
Time between diagnosis-discharge (weeks)	2 (0.9–7.1)	0.8 (0.5–2.7)	0.067
Maternal age	30.1 (26.4–34.2)	31.65 (26.65–36.4)	0.170
Age ≥ 35 years	19 (20.6)	17 (30.3)	0.182
Age ≥ 40 years	1 (1.1)	5 (8.9)	0.019
pBMI (kg/m^2^)	29.79 (25.09–33.73)	28.10 (25.90–33.39)	0.986
Overweight (BMI ≥ 25)	69 (75)	44 (78.6)	0.620
Obesity (BMI ≥ 30)	44 (47.8)	24 (42.9)	0.556
Grade II obesity (BMI ≥ 35)	17 (18.5)	13 (23.2)	0.487
Smoking	3 (3.26%)	4 (7.14%)	0.281
Chronic hypertension	1 (1.08%)	2 (3.57%)	0.303
Pre-gestational diabetes	2 (2.17%)	3 (5.35%)	0.299
Number of pregnancies	3 (1–3.5)	2 (2–3)	0.397
MAP (mmHg)	84.16 (80–90.5)	117 (96.33–124.33)	<0.001
MAP MoM	0.99 (0.90–1.06)	1.09 (0.96–1.15)	0.006
UtAm PI	0.75 (0.64–0.96)	0.73 (0.65–1.00)	0.506
MoM UtA	1 (0.87–1.21)	1.03 (0.91–1.28)	0.337
SpO_2_ (%)	93 (79.2–95.7)	93 (91–95)	0.537
Leukocytes (×10^9^/L)	8.5 (7.3–11.1)	9.65 (7.7–11.35)	0.384
Neutrophils (×10^9^/L)	6.6 (5.5–8.9)	7.25 (5.85–9.0)	0.528
Lymphocytes (×10^9^/L)	1.1 (0.8–1.4)	1.25 (0.9–1.65)	0.498
Hemoglobin (g/dL)	12.3 (11.2–13.3)	12.65 (11.6–14.3)	0.355
Platelets (×10^3^/L)	213 (185–269)	183 (145–230)	<0.001
Glucose (mg/dL)	79 (74–86)	80.5 (73–111)	0.473
Uric Acid (mg/dL)	4.25 (3.3–5.4)	5.8 (5.2–6.6)	0.002
ALT (U/L)	19 (12–35.5)	47 (26–78)	<0.001
AST (U/L)	23 (17–35)	36 (19–85)	<0.001
LDH (U/L)	188 (147–235)	225 (172–292)	0.052
Direct bilirubin (mg/dL)	0.11 (0.07–0.26)	0.15 (0.08–0.24)	0.515
Indirect bilirubin (mg/dL)	0.34 (0.27–0.46)	0.37 (0.27–0.54)	0.524
Triglycerides (mg/dL)	269 (204–332)	291 (226–352)	0.289
Cholesterol (mg/dL)	192 (158–233)	199 (135–237)	0.941
D-Dimer (ng/mL)	1,690 (1,262–2,953)	1,438 (1,009–4,083)	0.832
CPK (U/L)	38 (27–61)	45 (29–70)	0.563
CK-MB (U/L)	15.5 (11–21)	16 (15–23)	0.151
Troponin (ng/mL)	1.2 (0.3–2.1)	2.3 (1.1–5.7)	0.034
Myoglobin (ng/mL)	18.9 (13.2–31.1)	32.6 (18–54)	0.027
Procalcitonin (ng/mL)	0.1 (0.04–0.25)	0.22 (0.06–0.56)	0.146
C-RP (μg/mL)	24 (9.3–111)	69 (27–83)	0.394
Fibrinogen (mg/dL)	550 (481–600)	561 (486–638)	0.272
MoM PlGF	0.446 (0.231–0.688)	0.445 (0.212–0.815)	0.597
MoM sFlt-1	0.789 (0.545–1.758)	2.47 (1.00–5.22)	0.0001
sFlt-1/PlGF ratio	15.96 (6.425–53.095)	189.00 (63.89–489.02)	<0.001

*HDP, Hypertensive disease of pregnancy; pBMI, Pregestational body mass index; MAP, Mean arterial pressure; UtAm PI, Uterine artery pulsatility index; MoM, multiples of the median; SpO_2_, Oxygen saturation; AST, Aspartate aminotransferase; ALT, Alanine aminotransferase; LDH, Lactate dehydrogenase; CPK, Creatinine Phosphokinase; CK-MB, Creatine kinase; C-RP, C reactive protein; PlGF, Placental growth factor; sFlt-1, Soluble fms-like tyrosine kinase-1.*

*Mann-Whitney-U-test for continuous variables expressed as median and interquartile range (IQR); X^2^ or Fisher's test for categorical variables and expressed as numbers and percentages*.

**Table 2:** Maternal, fetal, and neonate outcomes for SARS-CoV-2 participants.

**Characteristic**	**Controls** **(*n* = 92)**	**HDP** **(*n* = 56)**	***p*-value**
**Maternal outcomes**			
Pneumonia	21 (22.8)	37 (66.1)	<0.001
Acute kidney injury	1 (1.1)	6 (10.7)	0.012
Multiple organ failure	3 (3.3)	0	1
Sepsis	6 (6.5)	0	0.591
Maternal death	5 (5.4)	0	0.157
Preterm rupture of membranes	5 (5.4)	2 (3.6)	0.470
Threatened preterm labor	6 (6.5)	0	0.591
UCI admission	11 (12)	5 (8.9)	0.160
Composite adverse maternal outcome	26 (28.3)	39 (69.6)	<0.001
**Fetal and neonatal outcomes**			
Gestational age at admission	33.35 (26.85–37.5)	33.55 (31.07–37.1)	0.219
Fetal-growth-restriction	9 (9.8)	3 (5.4)	0.540
Intrapartum fetal distress	7 (7.6)	0	0.348
Stillbirth	2 (2.2)	1 (1.8)	0.503
Gestational age at birth	38 (36–39)	38 (31–39)	0.760
Birthweight	2,890 (2,510–3,200)	2,020 (1,062–3,200)	0.026
Birthweight percentile	25 (0–54)	12 (0–33)	0.075
Apgar score	9 (9–9)	9 (8–9)	0.225
Low apgar	2 (2.2)	3 (5.4)	0.093
Intraventricular hemorrhage IV	2 (2.2)	2 (3.6)	0.238
Respiratory distress syndrome	9 (9.8)	4 (7.1)	0.516
NICU	6 (6.5)	2 (3.6)	0.881
Neonatal sepsis	17 (18.5)	6 (10.7)	0.755
Neonatal death	4 (4.3)	7 (12.5)	0.209
Composite adverse perinatal outcome	40 (43.5)	20 (35.7)	0.351

*HDP, Hypertensive disease of pregnancy; ICU, Intensive care unit; NICU, Neonatal intensive care unit; Mann-Whitney-U-test for continuous variables expressed as median and interquartile range; X^2^ or Fisher's test for categorical variables and expressed as numbers and percentages Mann-Whitney-U-test for continuous variables expressed as median and interquartile range; X^2^ or Fisher's test for categorical variables and expressed as numbers and percentages*.

### Association Between sFlt-1 and Composite Adverse Maternal Outcomes

According to AIC and BIC criteria, the best predictive model comprised sFlt-1 MoMs, HDP, ALT values, MAP, and sFlt-1/PlGF ratio (Table 3). After adjusting for each other, sFlt-1 MoM, HDP, the interaction between sFlt-1 MoM/HDP, and MAP were the only significant predictors of a composite of adverse maternal outcomes (Naeguelkerke *R*^2^: 0.42).

**Table 3:** Multivariate factorial model for adverse maternal outcome.

	**Odds ratio**	**95% CI**	***p*-value**
sFlt-1 MoM	5.13	2.19–12.05	<0.000
HDP	32.76	5.24–205	<0.000
Interaction sFlt-1/HDP	0.28	0.11–0.70	0.006
ALT	1.00	1.00–1.01	0.262
MAP	0.96	0.92–0.99	0.030
sFlt/PlGF ratio	1.00	1.00–1.00	0.151

*sFlt-1, Soluble fms-like tyrosine kinase-1; MoM, Multiples of the median; HDP, Hypertensive disease of pregnancy; ALT, Alanine aminotransferase; MAP, Mean arterial pressure; PlGF, Placental growth factor; CI, Confidence interval*.

**Figure 1:**
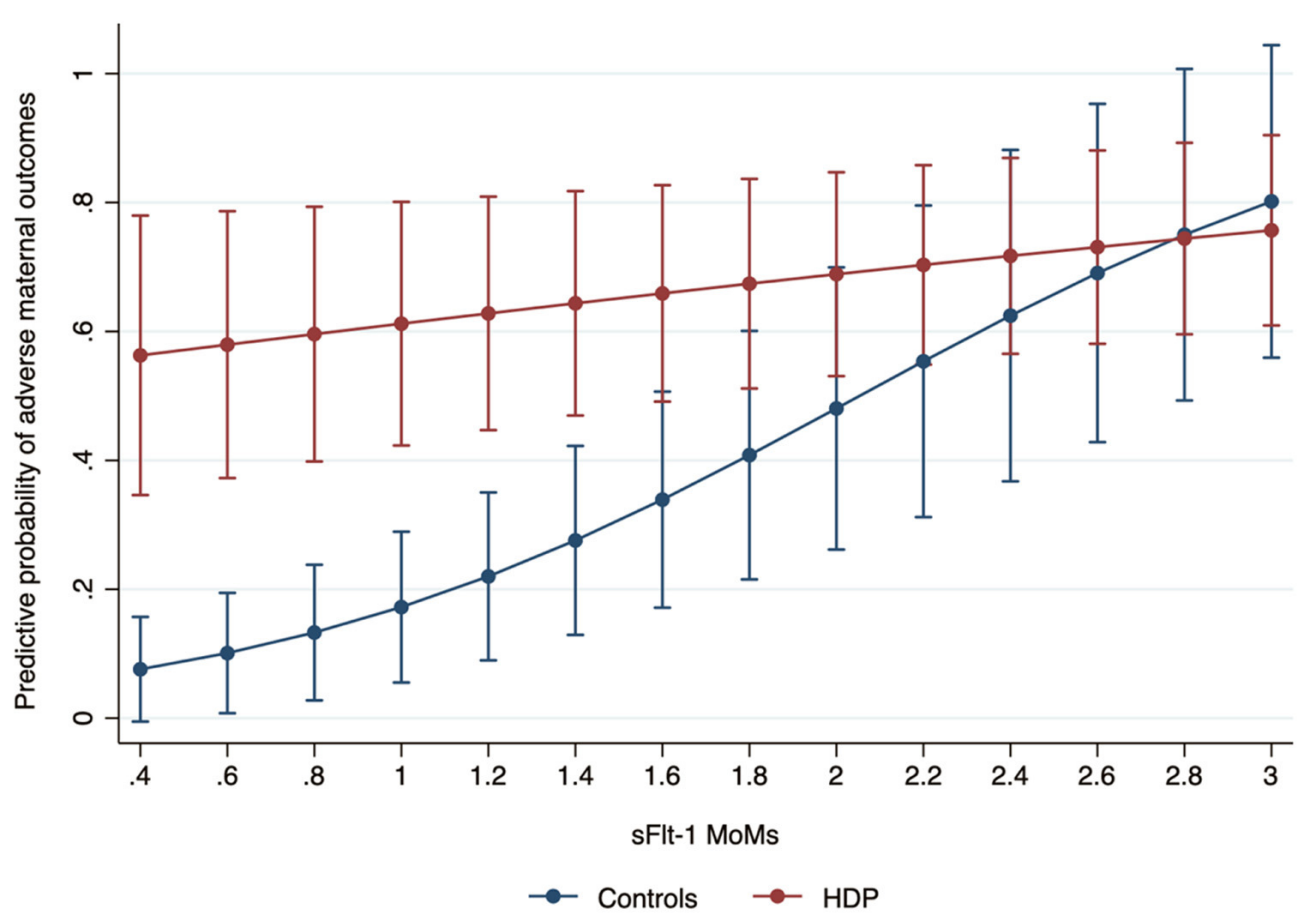
Predictive probability of an adverse maternal outcome among women with hypertensive disease of pregnancy (HDP) according to sFlt-1 MoM levels.

We observed a different correlation between sFlt-1 and AMO probability when comparing HDP and control groups. At low concentrations of sFlt-1, we found a significantly different risk for adverse maternal outcomes between the study groups (at 0.4 MoMs, 9 vs. 58%), as sFlt-1 concentrations increase, the difference in the probability of AMO decreases between groups; this equals at 2.8 multiples of the median (75%). Higher levels of sFlt-1 were independently associated with an increased probability of adverse maternal outcomes in both groups, but, as shown in Figure 1, the predictive capability is better in women without HDP as in this group a significant increase in the probability of adverse events as sFlt-1 levels increase (from 9% at 0.4 MoMs to 80% at 3 MoMs). This is not the case in women with HDP in whom the risk for adverse events is slightly increased as marker concentrations increase (from 59% at 0.4 MoMs to 77% at 3 MoMs). While in the group of patients with HDP the concentration of sFlt-1 does not adequately discriminate the risk of adverse maternal events, in the group of women without HDP its usefulness in identifying this risk is notable (Figure 1).

## Discussion

### Main Findings

The main finding of this study is that sFlt-1 is an independent predictor of adverse maternal outcomes after adjusting for confounders. Also, the probability of an adverse outcome increases as sFlt-1 MoM levels increase in women without HDP.

### Comparison With Existing Literature

Several studies have shown an additive adverse effect when COVID-19 and PE coexist (25–27). A recent meta-analysis described that SARS-CoV-2 infection during pregnancy increases the risk of preeclampsia (28).

In preeclamptic women, sFlt-1 levels and sFlt-1/PlGF ratio are higher in complications, such as pulmonary edema, acute renal failure, cerebral hemorrhage, and stillbirth (29, 30). In the present study, we found that pregnant patients complicated with SARS-CoV-2 infection-related hypertensive disorders of pregnancy have higher plasma levels of sFlt-1 than SARS-CoV-2 infection without HDP. SARS-CoV-2 infection initiates in the upper respiratory tract, using the transmembrane protein ACE-2 of host cells (31). However, the kidney, liver, heart, and placenta also express the ACE-2 protein (32). The viral components within endothelium and accumulation of inflammatory cells may lead to multi-organic endothelitis, because of excessive host response (11, 33–36).

In our population, SARS-CoV-2 infection is an independent inflammation factor, added to the pre-existing due to preeclampsia; the significant increases in sFlt-1 concentrations can explain this compared with women with SARS-CoV-2 infection without preeclampsia. Previously, we suggested that interaction between viral spike protein (S) of SARS-CoV-2 and ACE-2 reduces ACE-2 levels in the cell membrane and generates an imbalance of Ang-II/Ang1-7, causing acute hypoxia, which induction and overproduction of sFlt-1, leading to systemic endothelial damage (15, 37).

We suggest that increased sFlt-1 levels in our patients are due to the synthesis by other organs and tissues than the placenta, and the rising of complications in pregnancy patients positive for SARS-CoV-2 and hypertensive disorders; this can be explained by the presence of ACE-2 receptors in kidney, facilitating invasion and viral replication, that culminate in a direct injury, and increased proteinuria. So, upregulating ACE-2 expression in hepatocytes and the cholangiocytes leads to elevated liver enzymes (38, 39). This study provides new evidence that sFtl-1 is an independent marker of adverse maternal outcomes in women with COVID-19 after adjusting for hypertension in pregnancy.

### Strengths and Limitations of Study

The strengths of our study were that COVID-19 cases were consecutively recruited in a study with a specialized database built for research purposes, minimizing potential biases. In addition, baseline clinical characteristics between pregnant women with HDP and non-HDP were similar, diminishing the probability of selection bias.

Although sFlt-1 MoM has good performance in providing a short-term prediction of adverse maternal outcomes, the results need clinical validation in independent cohorts. Further evaluation of a risk cut-off for adverse maternal outcomes should be implemented in prospective cohorts.

### Clinical Interpretation

sFlt-1 levels could be used as a routine marker for all women with COVID-19 without hypertension at hospital admission. The risk of adverse maternal outcomes remains high among women with HDP complicated with COVID-19 and this justifies closer monitoring; surveillance should be implemented also in women with higher levels of sFlt-1 that do not present HDP at admission. A risk calculator could be helpful in the initial selection of women with COVID-19 to standardize conduct (Supplementary Material), while women with HDP should always be closely monitorized because of their higher risk for adverse events due to the interaction between hypertension and COVID-19.

## Conclusion

Our study shows that sFlt-1 is an independent predictor of adverse outcomes in women with SARS-CoV-2 despite hypertension status.

## Data Availability Statement

The original contributions presented in the study are included in the article/Supplementary Material, further inquiries can be directed to the corresponding author.

## Ethics Statement

The studies involving human participants were reviewed and approved by Ethics and Research Internal Review Board of the National Institute of Perinatology (2020-1-32). The patients/participants provided their written informed consent to participate in this study.

## Author Contributions

SE-y-S, JH-P, JT-T, and RM-P: study conception and design. PM-R, MN-T, ML-M, KE-S, NB-N, IC-R, MC-B, and MP-C: data collection. RM-P, GE-G, JV-B, and CM-M: analysis and interpretation of results. LR-Z and RM-P: statistical analysis. JH-P, JT-T, and SE-y-S: draft manuscript preparation. JS-P and AP-N: molecular studies. SE-y-S: oversight and leadership responsibility for the research activity planning and execution. JH-P and JT-T: management and coordination responsibility for the research activity planning and execution. All authors: reviewed the results and approved the final version of the manuscript.

## Conflict of Interest

The authors declare that the research was conducted in the absence of any commercial or financial relationships that could be construed as a potential conflict of interest.

## Publisher's Note

All claims expressed in this article are solely those of the authors and do not necessarily represent those of their affiliated organizations, or those of the publisher, the editors and the reviewers. Any product that may be evaluated in this article, or claim that may be made by its manufacturer, is not guaranteed or endorsed by the publisher.
